# Nutrition and immunity: lessons for COVID-19

**DOI:** 10.1038/s41430-021-00949-8

**Published:** 2021-06-23

**Authors:** Philip C. Calder

**Affiliations:** 1 School of Human Development and Health, Faculty of Medicine, University of Southampton, Southampton, UK; 2 NIHR Southampton Biomedical Research Centre, University Hospital Southampton NHS Foundation Trust and University of Southampton, Southampton, UK

**Keywords:** Nutrition, Infection

## Abstract

The role of the immune system is to protect the individual against pathogenic organisms. Nutrition is one of multiple factors that determines the immune response and good nutrition is important in supporting the immune response. Immunity can be impaired in older people, particularly those who are frail, in those living with obesity, in those who are malnourished and in those with low intakes of micronutrients. The immune impairments associated with nutritional inadequacy increase susceptibility to infection and permit infections to become more severe, even fatal. The adverse impact of poor nutrition on the immune system, including its inflammatory component, may be one of the explanations for the higher risk of more severe outcomes from infection with SARS-CoV-2 seen in older people and in those living with obesity. Studies of individual micronutrients including vitamin D and zinc suggest roles in reducing severity of infection with SARS-CoV-2. Good nutrition is also important in promoting a diverse gut microbiota, which in turn supports the immune system. The importance of nutrition in supporting the immune response also applies to assuring robust responses to vaccination. There are many lessons from the study of nutrition and immunity that are relevant for the battle with SARS-CoV-2.

## Introduction and scope

Coronaviruses are a large group of single-stranded RNA viruses that cause respiratory and, less frequently, gastrointestinal diseases. The respiratory symptoms caused by coronaviruses range from common cold-like or mild influenza-like symptoms to severe pneumonia. In December 2019, a new coronavirus causing pneumonia and death was identified in Wuhan, China; this new coronavirus is called severe acute respiratory distress syndrome coronavirus (SARS-CoV) 2 (SARS-CoV-2) because it is genetically similar to SARS-CoV which caused an outbreak of severe acute respiratory distress syndrome in 2002. Although SARS-CoV-2 is the seventh known human coronavirus, it is new to the human immune system and so there was no underlying existing immunity against it, explaining why SARS-CoV-2 spread so rapidly and has caused such severe illness; this illness is called coronavirus disease discovered in 2019 or COVID-19. The extent of the health, societal and economic consequences that have arisen due to the presence of SARS-CoV-2 and the severity of COVID-19 have focussed attention on the devastation infectious illness can cause and on the importance of having well-functioning immune systems. Inadequate immune responses have been exposed as a major public health liability in settings where this was previously either not well recognised or simply accepted. Vaccines work by training the immune system to work properly against a pathogen. The apparent effectiveness of the newly-developed vaccines to protect against COVID-19 is evidence of the inherent weakness of the immune system amongst significant subgroups in the population; nevertheless vaccinations themselves require a robust immune response to work properly and there has been some debate about the usefulness of some of the vaccines amongst those groups of the population who may have weakened immune responses. It is also likely that new vaccines will be developed in the future, especially with emergence of new variants of SARS-CoV-2. In the meantime, alongside the existing vaccination programmes, the development of new vaccines and the testing of new anti-viral drugs, it is important to consider other steps that can be taken to support the immune system. This article will describe the influence of ageing, frailty, obesity, micronutrients and the gut microbiota on the human immune system and discuss this in the contexts of SARS-CoV-2 infection and COVID-19.

## An overview of the role and organisation of the immune system

**Fig. 1:**
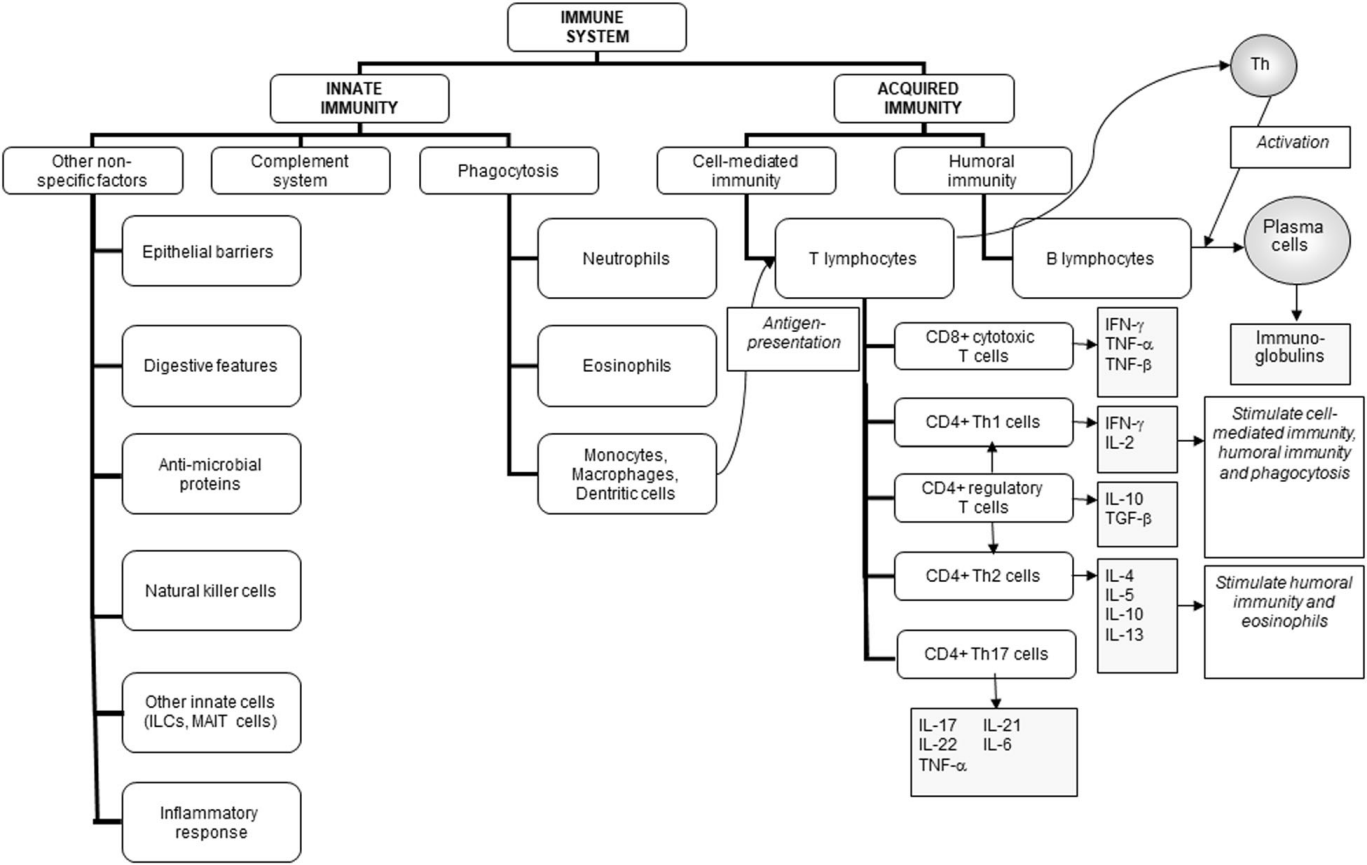
The components of the immune system and their division into innate and acquired immunity. IFN interferon, IL interleukin, ILCs innate lymphoid cells, MAIT mucosal associated invariant T, TGF transforming growth factor, TNF tumour necrosis factor.

**Fig. 2:**
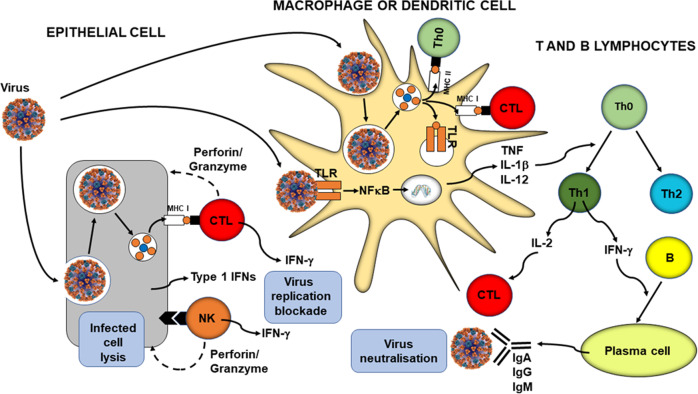
Overview of anti-viral immunity. B B-cell, CTL cytotoxic T-cell, IFN interferon, Ig immunoglobulin, IL interleukin, MHC major histocompatibility class, NFκB nuclear factor kappa-light-chain-enhancer of activated B cells, NK natural killer cell, Th helper T-cell, TLR toll-like receptor, TNF tumour necrosis factor. Taken from ref. [1].

The primary role of the immune system is to protect the individual against pathogenic organisms including bacteria, viruses, fungi and parasites. So that it can provide effective protection against the wide array of threatening organisms, the human immune system has evolved to include many different cell types, many communicating molecules and multiple functional responses (Fig. 1). The immune system has four general actions. Firstly, it acts as a barrier keeping microbes from entering the body. Secondly, the immune system acts to recognise microbes and to identify whether they are harmful or not. Thirdly, the immune system acts to eliminate those microbes identified as being harmful; this involves the destructive actions of various types of immune cell. Fourthly, the immune response generates immunological memory, so that if there is re-exposure to the harmful microbe, the immune response is more rapid and stronger than it was for the original response. These complex and sophisticated actions can be achieved because the human immune system is comprised of many cell types (Fig. 1), each with their own individual functional capabilities. These different cell types interact with one another as part of the immune response to assure effective protection of the host from pathogens. The immune system may be classified in different ways, most commonly into innate (or natural) and acquired (or adaptive) immunity (Fig. 1). Innate and acquired immunity are linked and how this is achieved to provide anti-viral immunity is summarised in Fig. 2 [1].

## Factors affecting the immune response

**Fig. 3:**
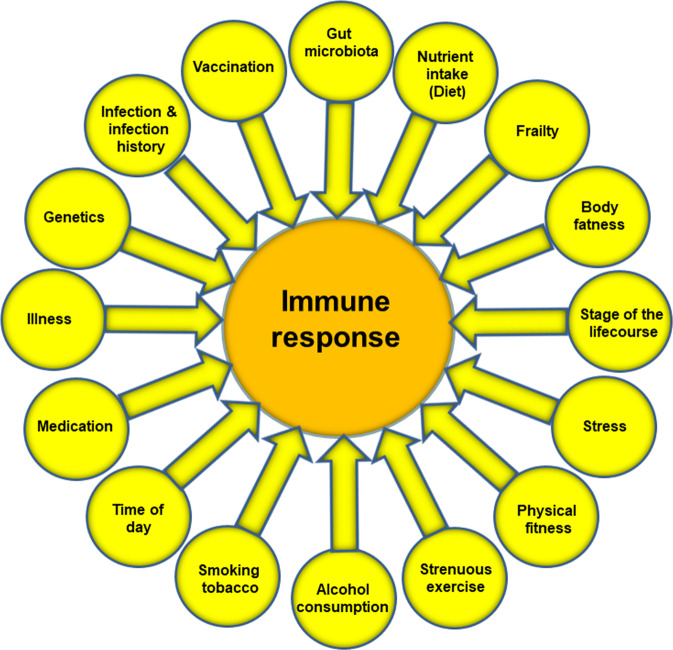
Factors that influence the immune response. Note that the listing is not exclusive.

It is obvious that effective defence against pathogenic organisms requires a well-functioning immune system. Consequently, individuals with weakened immune systems are at increased risk of becoming infected and of infections being more serious, even fatal. Figure 3 highlights many of the factors that influence the immune response. These include some unmodifiable factors such as genetics, stage of the life course (e.g. pregnancy, infancy and old age) and time of day, but many modifiable factors also influence the immune response. These include stress, physical fitness, frailty, body fatness and diet. Early in the SARS-CoV-2 pandemic it became clear that older people, particularly those who were frail, and that those living with obesity had higher susceptibility to more serious illness and mortality from COVID-19 than did younger people and those who were of healthy weight.

## The effect of ageing and frailty on immunity and susceptibility to infection

Immune competence can be diminished with ageing, a process called immunosenescence [2, 3]. One contributor to immunosenescence is likely to be the decreased output of immune cells from bone marrow, the site of origin of all immune cells, with increasing age. In addition, involution of the thymus with age decreases output of naive T cells, resulting in reduced capacity to respond to new antigens. In addition to altered numbers of immune cells in the circulation, their function is often impaired. For example, neutrophils show impaired phagocytosis, respiratory burst and bacterial killing. Natural killer cells have impaired cytotoxicity towards virally-infected and tumour cells. Dendritic cells have impaired responsiveness to immune signals. T cells have reduced ability to proliferate and to produce important cytokines like interleukin-2 and interferon-γ. Cytotoxic T-cell activity is reduced and antibody production by B cells is altered. Hence, older people can show a broad range of immune impairments, making them more susceptible to infections [4], including respiratory illnesses caused by viruses. Immunosenescence also impairs responses to vaccination, including to the seasonal influenza vaccine [5, 6]. Poor nutritional intake may contribute to age-related immune decline: immune decline is less in older people with better micronutrient intake or status [7]. Furthermore, amongst older people, undernutrition promotes immune decline [8] and frailty results in significant immune impairments. For example Yao et al. [9] reported that responses to all three strains within a seasonal influenza vaccine (responses measured as anti-vaccine antibody titres) were much lower in frail compared with non-frail older (72–95 years of age) people; responses of the pre-frail were intermediate. During a post-vaccination follow-up period, 50% of the frail older people developed influenza-like illness and 30% developed confirmed influenza; figures in the non-frail group were 10% and 5%, respectively, and again the pre-frail were intermediate between the frail and non-frail groups [9]. In a recent study, seroconversion of more frail older people to the four strains of a quadrivalent seasonal influenza vaccine was 8, 5, 0 and 8% while seroconversion in less frail older people was 23, 21, 23 and 26% [10]. That these immune impairments are of clinical significance comes from observations that less well-nourished hospitalised older people had a greater risk of infections than those who were better nourished [11, 12]. Thus, there is a link between immunosenescence and increased susceptibility to, and severity of, infections. Immunosenescence may be one factor that predisposes older people to more severe COVID-19. A number of studies also report a link between frailty and poorer outcome from COVID-19 (see ref. [13]). Ageing is also linked to an increase in blood concentrations of many inflammatory mediators, a situation termed inflammageing [14]. This state is considered to contribute to an increased risk of chronic conditions of ageing and may predispose to mounting an excessive inflammatory response when infected. Thus, older people, and again particularly those who are frail, may be pre-disposed to mounting an uncontrolled inflammatory response, sometimes termed a ‘cytokine storm’, that has been implicated in poor outcome from COVID-19. In summary, older people can show impaired immune responses, predisposing them to infection, and a proneness for uncontrolled inflammation, predisposing them to adverse consequences of being infected, and both these situations appear to be worse in those who are frail.

## The effect of obesity on immunity and susceptibility to infection

Immune competence can be diminished with obesity [15], with impairments of the activity of helper T cells, cytotoxic T cells, B cells and natural killer cells, and reduced antibody and interferon-γ production. This means that, compared with healthy weight individuals, those living with obesity have increased susceptibility to a range of bacterial, viral and fungal infections [16], and poorer responses to vaccination [17]. The impact of obesity has been well explored in relation to influenza infection and vaccination against influenza. During the 2009 H1N1 influenza A virus pandemic, those living with obesity showed delayed and weakened anti-viral responses to infection and showed poorer recovery from disease compared with healthy weight individuals [18]. Animal studies and case studies in humans show that obesity is associated with prolonged shedding of influenza virus, indicating an impairment in viral control and killing; obesity is also linked with the emergence of virulent minor variants [18]. Vaccines may also be less effective in those living with obesity: compared with healthy weight individuals, vaccinated individuals living with obesity have twice the risk of influenza or influenza-like illness, indicating poorer protection from vaccination in those with obesity [19]. Sheridan et al. [20] investigated the responses of immune cells taken from the blood of healthy weight individuals and those with overweight or obesity to the influenza vaccine in vitro. Exposure of the blood immune cells to the vaccine increased the number of activated cytotoxic T cells, the number of granzyme expressing cytotoxic T cells and the number of interferon-γ producing cytotoxic T cells, all key components of anti-viral immunity (Fig. 2). However, the responses of cells from individuals with obesity were reduced by 40%, almost 60% and 65%, respectively. Cells from individuals with overweight showed responses intermediate between those from healthy weight and those with obesity. Similar findings for the response of blood cells to the pandemic H1N1 influenza A virus were reported by Paich et al. [21]. Thus, obesity is linked to multiple immune impairments, including to responses involved in protection against viruses. Obesity is also associated with an increase in blood concentrations of many inflammatory mediators, a state of chronic low-grade inflammation [22]. This state is considered to contribute to an increased risk of chronic conditions associated with obesity and may predispose to mounting an excessive inflammatory response when infected. It is now well described that those living with obesity are more susceptible to severe COVID-19 and to mortality from COVID-19 than healthy weight adults. For example, a recently published systematic review and meta-analysis of 22 studies from seven countries in North America, Europe, and Asia, reported that obesity is associated with an increased likelihood of presenting with more severe COVID-19 symptoms (odds ratio 3.03; four studies), requiring hospitalisation (adds ratio 1.68; four studies), being admitted to an intensive care unit (odds ratio 1.35; nine studies), undergoing invasive mechanical ventilation (odds ratio 1.76; seven studies) and developing acute respiratory distress syndrome (odds ratio 2.89; two studies) compared to patients without obesity [23]. In summary, those living with obesity can show impaired immune responses, predisposing them to infection, and a proneness towards uncontrolled inflammation, predisposing them to adverse consequences of being infected.

## The role of micronutrients in supporting the immune response

Nutrition plays multiple roles in supporting the immune system. The diet provides:

- Fuels for the immune system to function.
- Building blocks for the generation of RNA and DNA and for the production of proteins (antibodies, cytokines, receptors, acute phase proteins etc.) and new cells.
- Specific substrates for the production of immune-active metabolites (e.g. arginine as a substrate for nitric oxide).
- Regulators of immune cell metabolism (e.g. vitamin A, zinc).
- Nutrients with specific antibacterial or anti-viral functions (e.g. vitamin D, zinc).
- Regulators that protect the host from oxidative and inflammatory stress (e.g. vitamin C, vitamin E, zinc, selenium, long-chain omega-3 fatty acids and many plant polyphenols).
- Substrates for the intestinal microbiota which in turn modulates the immune system (see next section).

**Fig. 4:**
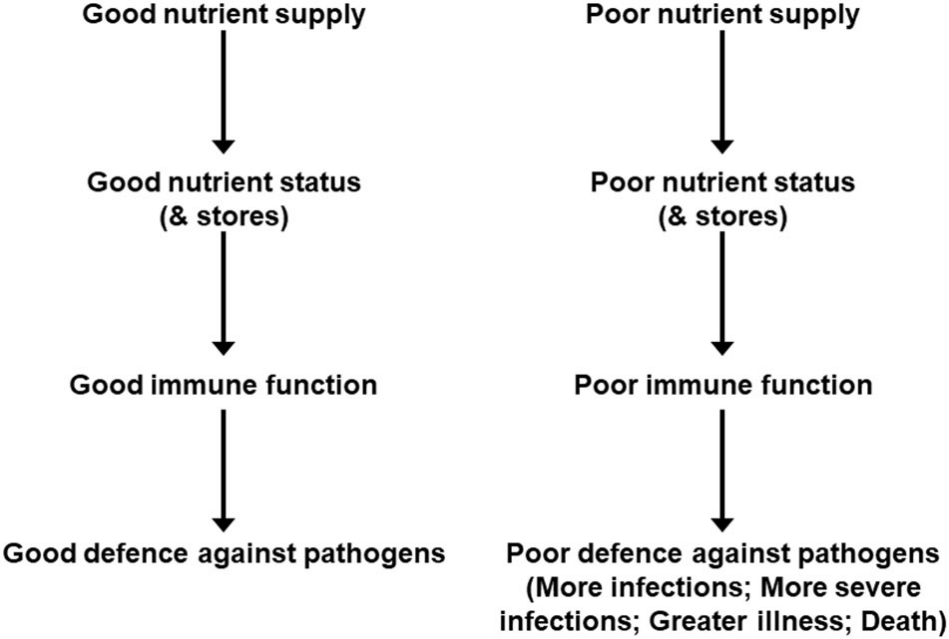
Relationships between good and poor nutrition, immunity and infection.

Poor nutrition may not provide sufficient amounts of the nutrients needed by the immune system to function well. This would be associated with increased susceptibility to infection and inability to control the effects of being infected (Fig. 4). In this regard the role of micronutrients in supporting the immune system has been widely studied, as reviewed elsewhere [1, 24–26]. Multiple micronutrients play vital roles in supporting the immune response (Table 1). The roles of vitamins A, C and D and zinc, copper and iron are well explored, but B vitamins, vitamin E, vitamin K, selenium, magnesium and others all have roles. Deficiencies of several of these micronutrients impair many aspects of both innate and acquired immunity and increase susceptibility to infections [1, 24]. The immune impairments can be reversed by repletion and this reduces susceptibility to infection. There has been discussion around many micronutrients and anti-viral immunity in the context of infection with SARS-CoV-2 and COVID-19 and there have been numerous publications on this topic since the start of the SARS-CoV-2 pandemic.

**Table 1:** Summary of the effects of various micronutrients on different aspects of immunity.

Micronutrient	Role in barrier function	Role in cellular aspects of innate immunity	Role in T-cell mediated immunity	Role in B-cell mediated immunity
Vitamin A	Promotes differentiation of epithelial tissue; promotes gut homing of B- and T cells; promotes intestinal immunoglobulin A^+^ cells; promotes epithelial integrity	Regulates number and function of NK cells; supports phagocytic and oxidative burst activity of macrophages	Regulates development and differentiation of Th1 and Th2 cells; promotes conversion of naive T cells to regulatory T cells; regulates IL-2, IFN-γ and TNF production	Supports function of B cells; required for immunoglobulin A production
Vitamin B6	Promotes gut homing of T cells	Supports NK-cell activity	Promotes T-cell differentiation, proliferation and function, especially Th1 cells; regulates (promotes) IL-2 production	Supports antibody production
Vitamin B9 (Folate)	Survival factor for regulatory T cells in the small intestine	Supports NK-cell activity	Promotes proliferation of T cells and the Th1-cell response	Supports antibody production
Vitamin B12	Important co-factor for gut microbiota	Supports NK-cell activity	Promotes T-cell differentiation, proliferation and function, especially cytotoxic T cells; controls ratio of T-helper to cytotoxic T cells	Required for antibody production
Vitamin C	Promotes collagen synthesis; promotes keratinocyte differentiation; protects against oxidative damage; promotes wound healing; promotes complement	Supports function of neutrophils, monocytes and macrophages including phagocytosis; supports NK-cell activity	Promotes production, differentiation and proliferation of T cells especially cytotoxic T cells; regulates IFN-γ production	Promotes antibody production
Vitamin D	Promotes production of antimicrobial proteins (cathelicidin, β-defensin); promotes gut tight junctions (via E-cadherin, connexion 43); promotes homing of T cells to the skin	Promotes differentiation of monocytes to macrophages; promotes macrophage phagocytosis and oxidative burst	Promotes antigen processing but can inhibit antigen presentation; can inhibit T-cell proliferation, Th1-cell function and cytotoxic T-cell function; Promotes the development of regulatory T cells; inhibits differentiation and maturation of dendritic cells; regulates IFN-γ production	Can decrease antibody production
Vitamin E	Protects against oxidative damage	Supports NK-cell activity	Promotes interaction between dendritic cells and T cells; promotes T-cell proliferation and function, especially Th1 cells; regulates (promotes) IL-2 production	Supports antibody production
Zinc	Maintains integrity of the skin and mucosal membranes; promotes complement activity	Supports monocyte and macrophage phagocytosis; supports NK-cell activity	Promotes Th1-cell response; Promotes proliferation of cytotoxic T cells; promotes development of regulatory T cells; regulates (promotes) IL-2 and IFN-γ production; reduces development of Th9 and Th17 cells	Supports antibody production particularly immunoglobulin G
Copper		Promotes neutrophil, monocyte and macrophage phagocytosis; supports NK-cell activity	Regulates differentiation and proliferation of T cells; regulates (promotes) IL-2 production	
Iron	Essential for growth and differentiation of epithelial tissue	Promotes bacterial killing by neutrophils; regulates balance of M1 and M2 macrophages; supports NK-cell activity	Regulates differentiation and proliferation of T cells; regulates IFN-γ production	
Selenium		Supports NK-cell activity	Regulates differentiation and proliferation of T cells; regulates (promotes) IFN-γ production	Supports antibody production

*IFN* Interferon, *IL* interleukin, *NK* natural killer, *Th* T-helper, *TNF* tumour necrosis factor.

Vitamin D has pleiotropic actions within the immune system but does support the activity of several cell types [27]. Furthermore, some immune cells (dendritic cells, macrophages) can produce the active form of vitamin D suggesting it is important to immunity. Vitamin D also promotes the production of antimicrobial proteins such as cathelicidin. Vitamin D deficiency impairs the response to the seasonal influenza vaccine [28] and meta-analyses of randomised controlled trials of vitamin D supplementation report reduced incidence of respiratory tract infections [29]. Multiple studies report an association between low vitamin D status and increased susceptibility to, and severity of, COVID-19. A large Israeli study reported that low vitamin D status increased the risk of infection with SARS-CoV-2 and increased risk of hospitalisation with COVID-19 [30]. Meta-analyses report that vitamin D deficiency increases risk of severe COVID-19, hospitalisation with COVID-19 and mortality from COVID-19 [31]. A large study using data from the UK Biobank reported that using vitamin D supplements decreased risk of a positive test for SARS-CoV-2 after controlling for multiple confounders [32]. A study in an Italian residential care home reported that a bolus of vitamin D reduced mortality from COVID-19 [33]. Vitamin D supplementation in patients hospitalised with COVID-19 is reported to reduce COVID-19 severity (need for intensive care unit admission [34]; need for intensive care unit admission or mortality [35]; mortality [36]).

Zinc supports the activity of many cells of the immune system [37], helps to control oxidative stress and inflammation and has specific anti-viral actions [38] including inhibiting the replication of coronaviruses [39]. Zinc supplementation improves some markers of immunity especially in older people or those with low zinc intake [40], improves vaccination responses [41] and meta-analyses of randomised controlled trials of zinc supplementation report reduced incidence of diarrhoeal and respiratory tract infections (see ref. [1] for references). Multiple studies report an association between low zinc status and increased susceptibility to and severity of COVID-19 (e.g. [42]). Zinc supplementation in patients hospitalised with COVID-19 is reported to reduce risk of poor outcome including mortality [43, 44].

In contrast to the large literature on vitamin D and zinc that has emerged during the pandemic, there has been less research on selenium. Nevertheless, selenium may have important roles in supporting the immune system in general and in promoting anti-viral immunity in particular [45]. Selenium supports the activity of many cells of the immune system and helps to control oxidative stress and inflammation. Extensive research in mice has shown that selenium deficiency impairs immune responses, increases susceptibility to viral infection, permits viruses (including influenza viruses) to mutate, and allows normally weak viruses to become more virulent. Selenium supplementation improves some markers of immunity especially in older people or those with low selenium intake; for example a supplementation study conducted in UK adults with marginal selenium status showed that selenium improved ex vivo anti-viral immune responses, promoted viral clearance and decreased viral mutation [46]. Several studies report an association between low selenium status and increased susceptibility to and severity of COVID-19 (e.g. [42, 47]).

Taken together, the existing evidence indicates that multiple micronutrients play vital roles in supporting all aspects of the immune response and therefore that their intake and status need to be considered in the context of susceptibility to SARS-CoV-2 infection and COVID-19 severity. Roles of specific nutrients including vitamin D and zinc in anti-viral immunity seem to be important and the ability of selenium to prevent viral mutation is intriguing in the context of the emergence of SARS-CoV-2 variants. Furthermore, low intakes of several micronutrients impair vaccination responses and so must be considered in the context of the current and future COVID-19 vaccination programmes; this is likely to be particularly important in the elderly [48] but also in other groups who are more likely to have low intakes or status of one or more micronutrients. Although micronutrients are provided as part of a diverse, plant-based diet (see ref. [1]) there is a question about whether sufficient amounts of some of the key immune-active micronutrients (vitamin D, vitamin C, vitamin E, zinc and selenium) can be obtained from the diet or whether supplements are necessary to provide the relevant intakes of these micronutrients [26].

## The importance of the gut microbiota

Commensal bacteria within the gastrointestinal tract play a role in host immune defence by creating a barrier against colonisation by pathogens and through the production of lactic acid and antimicrobial proteins which can directly inhibit the growth of pathogens. Commensal organisms also interact with the host’s gut epithelium and gut-associated immune tissues. These communications with the host occur through chemicals released from the bacteria or through direct cell-to-cell contact. As a result of such actions, it is proposed that probiotic organisms, particularly some lactobacilli and bifidobacteria, can be used to support host immunity. In fact, a large number of studies have examined the influence of various probiotic organisms, either alone or in combination, on immune function and infection in human subjects. Some probiotic organisms appear to enhance innate immunity (particularly phagocytosis and natural killer cell activity) but they seem to have a less pronounced effect on acquired immunity [49]. Nevertheless, studies show improved vaccination responses in individuals taking probiotics, as reviewed elsewhere [50]. Systematic reviews and meta-analyses confirm that probiotics (or prebiotics) enhance the antibody response to seasonal influenza vaccination in adults [51, 52]. The immune effects observed suggest that probiotic organisms could protect against infections. Recent systematic reviews and meta-analyses report that some probiotics can reduce the risk or duration of diarrhoea, including antibiotic-associated diarrhoea and *Clostridium difficile*-associated diarrhoea (see ref. [1] for references). Effects of probiotics on gastrointestinal infection may not be a surprise, but probiotics may also be protective against respiratory infection. Studies in mice have reported that depletion or absence of gut microbiota leads to impaired immune responses and worsen outcomes following bacterial or viral respiratory infection. Studies of probiotics, particularly lactobacilli and bifidobacterial, provide some evidence for reduced incidence, and improved outcomes, of respiratory infections in humans (see ref. [1] for references). The totality of the evidence demonstrating that probiotics (especially lactobacilli and bifidobacteria) may improve immune function, enhance the response to seasonal influenza vaccination (which mimics a viral infection), reduce the incidence of respiratory infections, including those caused by viruses, and improve outcomes in those with respiratory infections would favour the use of these organisms as a strategy to reduce the risk and severity of viral respiratory infections, including SARS-CoV-2. In this context, intestinal dysbiosis, with low numbers of lactobacilli and bifidobacteria, has been reported in patients with COVID-19 [53, 54]. D’Ettore et al. [55] treated patients with COVID-19 with a cocktail of drugs plus antibiotics or the same plus oral probiotics (five lactobacilli plus two bifidobacteria plus *Streptococcus thermophilus*): they found better resolution of diarrhoea and of other disease symptoms including respiratory disease in the group receiving probiotics.

## Discussion and conclusions

**Fig. 5:**
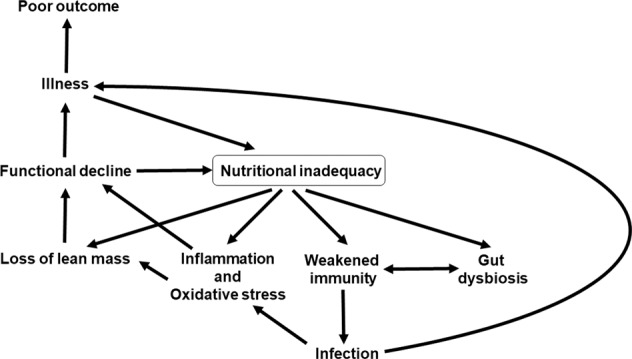
Factors linking nutritional inadequacy with infection and poor outcome from infection.

Nutrition is one of multiple factors that determines the immune response (Fig. 3) and good nutrition is important in supporting the immune response (Fig. 4). Immunity can be impaired in older people, particularly those who are frail, in those living with obesity, in those who are malnourished and in those with low intakes of micronutrients. These immune impairments associated with nutritional inadequacy increase susceptibility to infection and permit infections to become more severe, even fatal (Fig. 5). Nutritional inadequacy also allows dysregulated inflammation and oxidative stress contributing to frailty and to poor outcome from infection (Fig. 5). The adverse impact of poor nutrition on the immune system, including its inflammatory component, may be one of the explanations for the higher risk of more severe outcomes from infection with SARS-CoV-2 seen in older people and in those living with obesity. The role of good nutrition in promoting a diverse gut microbiota, which in turn supports the immune system should not be overlooked and it is important to note that the gut microbiota is also affected by ageing and by obesity (see [1] for references). The importance of good nutrition in supporting the immune response also applies to assuring good responses to vaccination. Thus, attention should be focussed on addressing the current nutritional inadequacies (frailty, obesity, general undernutrition, micronutrient insufficiency or deficiency) that are widespread in the population in order to better support the immune response. This is the major lesson from the study of nutrition and immunity that is relevant for the battle with SARS-CoV-2 and the disease it causes, COVID-19, and for ensuring the population is better prepared for future pandemics.
